# F18-FDG PET/CT imaging early predicts pathologic complete response to induction chemoimmunotherapy of locally advanced head and neck cancer: preliminary single-center analysis of the checkrad-cd8 trial

**DOI:** 10.1007/s12149-022-01744-6

**Published:** 2022-05-10

**Authors:** M. Beck, J. Hartwich, M. Eckstein, D. Schmidt, A. O. Gostian, S. Müller, S. Rutzner, U. S. Gaipl, J. von der Grün, T. Illmer, M. G. Hautmann, G. Klautke, J. Döscher, T. Brunner, B. Tamaskovics, A. Hartmann, H. Iro, T. Kuwert, R. Fietkau, M. Hecht, S. Semrau

**Affiliations:** 1 Clinic of Nuclear Medicine, Friedrich-Alexander-Universität Erlangen-Nürnberg, University Hospital Erlangen, Ulmenweg 18, 91054 Erlangen, Bayern Germany; 2 Institute of Pathology, Friedrich-Alexander-Universität Erlangen-Nürnberg, Erlangen, Bayern Germany; 3 Department of Otolaryngology-Head and Neck Surgery, Friedrich-Alexander-Universität Erlangen-Nürnberg, Erlangen, Bayern Germany; 4 Department of Radiation Oncology, Friedrich-Alexander-Universität Erlangen-Nürnberg, Erlangen, Bayern Germany; 5 Department of Radiotherapy and Oncology, Goethe University Frankfurt, Frankfurt am Main, Germany; 6 Medical Oncology Clinic Dresden Freiberg, Dresden, Saxony Germany; 7 Department of Radiotherapy, Universität Regensburg, Regensburg, Bayern Germany; 8 Department of Radiation Oncology, Chemnitz Hospital, Chemnitz, Sachsen Germany; 9 Department of Otolaryngology-Head and Neck Surgery, Universität Ulm, Ulm, Baden-Württemberg Germany; 10 Department of Radiation Oncology, Otto Von Guericke Universität Magdeburg, Magdeburg, Sachsen-Anhalt Germany; 11 Department of Radiation Oncology, Heinrich-Heine-Universität Düsseldorf, Düsseldorf, Nordrhein-Westfalen Germany

**Keywords:** Immunotherapy, HNSCC, Head neck cancer, FDG-PET/CT, Induction therapy

## Abstract

**Aim:**

In the CheckRad-CD8 trial patients with locally advanced head and neck squamous cell cancer are treated with a single cycle of induction chemo-immunotherapy (ICIT). Patients with pathological complete response (pCR) in the re-biopsy enter radioimmunotherapy. Our goal was to study the value of F-18-FDG PET/CT in the prediction of pCR after induction therapy.

**Methods:**

Patients treated within the CheckRad-CD8 trial that additionally received FDG- PET/CT imaging at the following two time points were included: 3–14 days before (pre-ICIT) and 21–28 days after (post-ICIT) receiving ICIT. Tracer uptake in primary tumors (PT) and suspicious cervical lymph nodes (LN +) was measured using different quantitative parameters on EANM Research Ltd (EARL) accredited PET reconstructions. In addition, mean FDG uptake levels in lymphatic and hematopoietic organs were examined. Percent decrease (Δ) in FDG uptake was calculated for all parameters. Biopsy of the PT post-ICIT acquired after FDG-PET/CT served as reference. The cohort was divided in patients with pCR and residual tumor (ReTu).

**Results:**

Thirty-one patients were included. In ROC analysis, ΔSUVmax PT performed best (AUC = 0.89) in predicting pCR (*n* = 17), with a decline of at least 60% (sensitivity, 0.77; specificity, 0.93). Residual SUVmax PT post-ICIT performed best in predicting ReTu (*n* = 14), at a cutpoint of 6.0 (AUC = 0.91; sensitivity, 0.86; specificity, 0.88). Combining two quantitative parameters (ΔSUVmax ≥ 50% and SUVmax PT post-ICIT ≤ 6.0) conferred a sensitivity of 0.81 and a specificity of 0.93 for determining pCR. Background activity in lymphatic organs or uptake in suspected cervical lymph node metastases lacked significant predictive value.

**Conclusion:**

FDG-PET/CT can identify patients with pCR after ICIT via residual FDG uptake levels in primary tumors and the related changes compared to baseline. FDG-uptake in LN + had no predictive value.

**Trial registry:**

ClinicalTrials.gov identifier: NCT03426657.

## Introduction

Squamous cell cancers of the head and neck region (HNSCC) are quite prevalent, representing the sixth common cancer entity. In patients with advanced non-resectable disease the 3-year progression-free survival is approximately 50–60% [1, 2] Surgery and/or combination radio- and chemotherapy (CRT) are standard therapeutic options often permanently impair quality of life [3]. The concept of short-term induction chemotherapy (IC) prior to radiotherapy has more recently become a controversial topic [4–8]. Although long-term IC did not improve overall survival (OS) [9], there is evidence that monitoring tumor response to a single cycle of IC helps identify patients best suited for laryngeal preservation with CRT [6]. Furthermore, F-18 fluorodeoxyglucose positron emission tomography/computed tomography (FDG-PET/CT) has proven useful in assessing metrics other than histologic response, correctly determining reduced tumor metabolism after IC [6, 10, 11] and predicting long-term outcomes after CRT [5]. This approach is superior to conventional imaging (i.e. CT) and visual endoscopic examination in sensitivity and specificity [7].

Immunotherapy (IT) with immune checkpoint inhibitors is a newer treatment modality used successfully in a rising number of malignancies [12, 13] and has become first-line therapy in recurrent/metastatic HNSCC [14]. The CheckRad-CD8 study is currently investigating a combined regimen of induction immunochemotherapy consisting of combined PD-L1/CTLA-4 checkpoint blockade followed by chemotherapy-free radioimmunotherapy for locally advanced HNSCC. Patient selection for radioimmunotherapy is based on the patients` responses after single cycle ICIT. Biopsy specimens and FDG-PET/CT studies are performed before and after ICIT.

At present, it is unclear whether FDG-PET/CT is a reasonable means of therapeutic monitoring in this early phase of therapy. IT agents, especially CTLA-4-inhibitors, cause substantial influx of immune cells into tumors, triggering localized inflammatory reactions [15]. Such changes appear as heightened metabolic foci on FDG-PET/CT scans and no doubt are of benefit when screening for inflammatory or infectious disease [16]. In this context, however, treatment-related metabolic changes may be entirely misleading (particularly during early-phase therapy) interpreted as insufficient therapeutic responses that prompt unnecessary changes in treatment [17]. Instances of pseudoprogression detected by CT and FDG-PET/CT in early phases of IT have been reported in the literature, undermining the diagnostic utility of these scans [17, 18].

Our goal was to prospectively investigate the use of FDG-PET/CT for therapeutic monitoring after ICIT in patients with locally advanced HNSCC. Endoscopy with biopsy of the primary tumor post-ICIT was acquired after FDG-PET/CT and served as reference. To our knowledge, this is the first attempt to investigate early ICIT responses in the above setting using quantifiable parameters of FDG-PET/CT.

## Materials and methods

### Trial design and treatment

CheckRad-CD8 is a multicenter open-label phase II study with a single treatment arm in patients with locally advanced HNSCC without distant metastases (UICC stage III to IVB, according TNM version 8) and with no prior treatment. Patients received a single cycle of ICIT in week 1 with cisplatin 30 mg/m^2^ body surface area (BSA) on days 1–3 and docetaxel 75 mg/m^2^ BSA on day 1. The immune checkpoint inhibitors tremelimumab (anti-CTLA4) at a fixed dose of 75 mg and durvalumab (anti-PDL1) at a fixed dose of 1500 mg were both administered on day 5. Restaging was performed in week 4 with endoscopy with representative re-biopsy of the primary tumor. Patients with increased intratumoral CD8 + cell density (at least + 20% compared to baseline) or pCR continued with radioimmunotherapy. To ensure a sufficient coverage of a former tumor bed, a relevant resorptive inflammation together with granulation and scar tissue was required in the pathologic assessment of post therapeutic biopsies. Radiotherapy was performed as intensity modulated radiation therapy (IMRT) with a simultaneous integrated boost. A dose per fraction of 2.0/1.8/1.6 Gy was delivered up to 70.0/63.0/56 Gy in 35 fractions. Immunotherapy was continued with two concomitant and one subsequent doses of combined durvalumab and tremelimumab followed by eight doses of durvalumab maintenance therapy administered every fourth week.

**Fig. 1:**
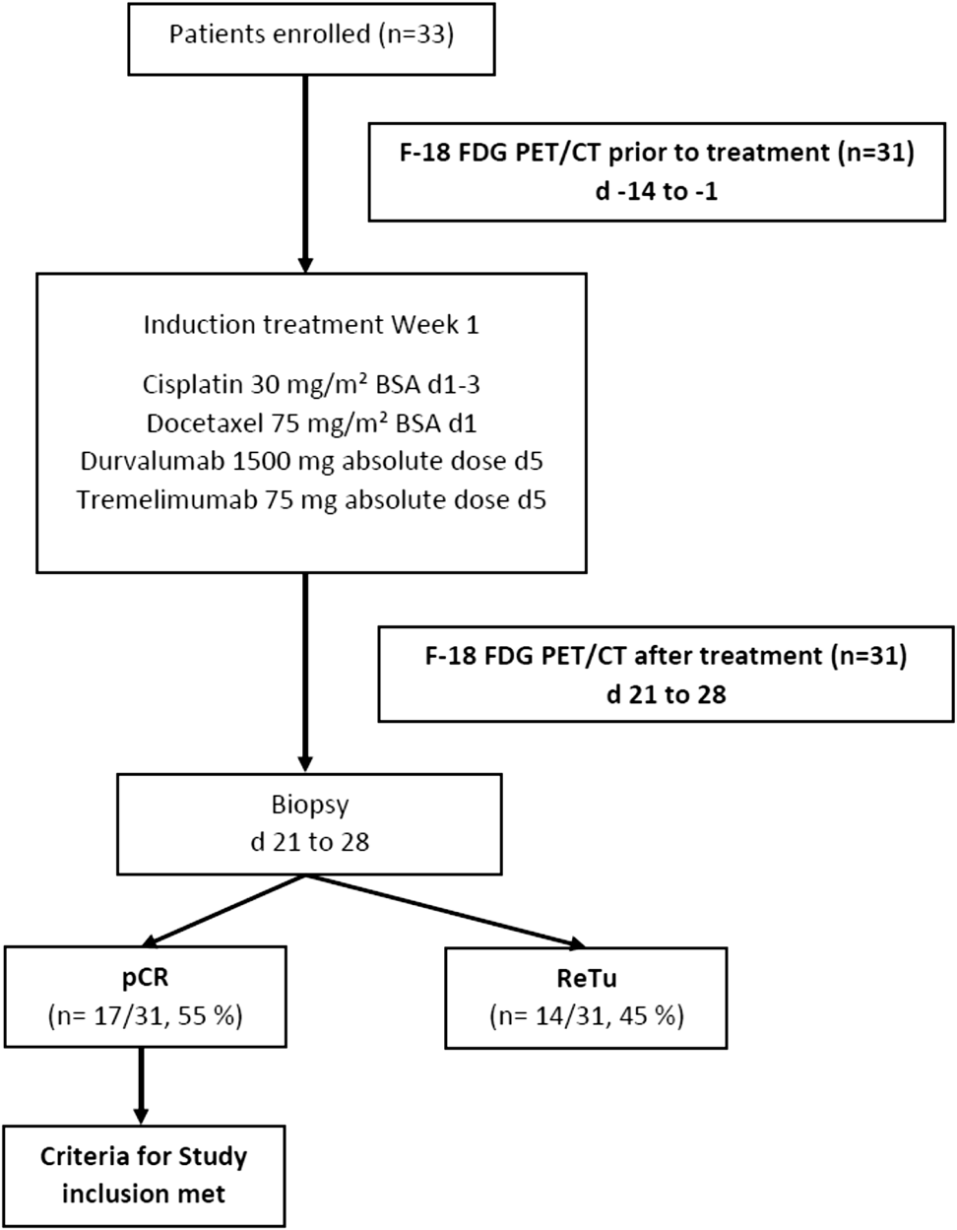
Flowchart of the CheckRad CD-8 trial design *BSA* body surface area, *pCR* pathological complete remission; *ReTu* residual tumor

Patients eligible for this analyses were scanned 1–14 days before (pre-ICIT) and 21–28 days after (post-ICIT) ICIT initiation via FDG-PET/CT. PET scans were only performed in the subgroup of patients treated in one study center. A flowchart of the study can be found in Fig. 1.

### Trial oversight

The trial was registered with ClinicalTrials.gov (identifier: NCT03426657). The local review board (number: 131_18 Az) approved the trial. All patients gave written informed consent. The trial was funded by AstraZeneca (ESR-16-12,356) and was conducted as investigator sponsored trial.

### Re-biopsy

Restaging was performed in week 4 with representative re-biopsy of the primary tumor. The re-biopsy was performed in mean 3.3 ± 2.6 days after the post-ICIT PET scan. Biopsy was planned according to the results of the PET. Samples out of the hottest regions of the tumor bed were gathered.

### Scanning procedures

FDG-PET/CT imaging was performed using a Biograph mCT scanner (Siemens Healthineers, Erlangen, Germany), with a field of view of 21.8 cm. Patients fasted for at least 4 h prior to procedures. Blood sugar levels in routine testing were < 180 mg/dl in all patients. Data acquisition began on average 60 min (± 10 min) after injecting ~ 3 MBq/kg F-18-FDG, scanning patients for 3 min in each bed position from the head to the mid thighs. PET data were reconstructed using a European Association of Nuclear Medicine (EANM) Research Ltd (EARL) criteria v1.0- accredited iterative time-of-flight (TOF) reconstruction method (256 × 256 matrix, voxel size 3.2 × 3.2 × 2 mm) with 21 iterations and one subset, without correcting for point spread function. A 6-mm Gaussian filter was applied. Additionally, a diagnostic, contrast enhanced CT, covering the same area, was performed (120 kV tube voltage, 160 mAs tube current with patient individual modulation CAREDose4D (Siemens Healthineers, Erlangen, Germany)). CT data were reconstructed using a filtered back projection method with a slice thickness of 1.5 mm and B41 and B70 kernel.

### Outcome measures

Two physicians specializing in nuclear medicine and skilled in interpreting PET scans, blinded for the clinical information, reviewed all studies, both visually and quantitatively. All reviews took place on dedicated workstations using proprietary software (syngo.via; Siemens Healthineers). Lesions with questionable tracer uptake were rated by consensus. On baseline and post-ICIT PET scans (pre-ICIT and post-ICIT), tracer uptake levels at primary tumor (PT) sites and within suspicious cervical lymph nodes (LN +) were measured as body weight-corrected standardized uptake values (SUVs). The following quantitative parameters were assessed by isocontour volumes of interest (VOIs) at thresholds of 40%: SUVmax PT, SUVpeak PT, metabolic tumor volume (MTV) PT, total lesion glycolysis (TLG) PT, SUVmax LN + , SUVpeak LN + , MTV LN + , TLG LN + , whole-body (WB) MTV, and WB TLG. For the measurement of SUVmax LN + and SUVpeak LN + the hottest LN + on the pre-ICIT PET scan was selected. The same measurements were then also performed in the post-ICIT PET scan on this LN + . For WB TLG and WB MTV the TLG and MTV of all suspicious lesions were added up.

In addition, the SUVmean of liver, spleen, vertebral bodies L1-L3, and infracarinal lymph node region 7 were measured using spherical VOIs. These regions were selected as representative organs of the lymphatic and the hematopoietic system, possibly monitoring the immune reaction induced by immunotherapy.

Declines in metabolic activity were calculated as follows:

% Activity Decline (eg, ΔSUVmax PT) = (SUVmax PT pre-ICIT–SUVmax PT post-ICIT)/SUVmax PT pre-ICIT × 100.

Prior to and 21–28 days after ICIT initiation, tumors were also subjected to endoscopic biopsy, collecting representative samples. The cohort was divided in patients with pCR and residual tumor (ReTu). To gauge sensitivities, specificities, positive predictive values (PPVs), and negative predictive values (NPVs) of quantitative assessments, patients were grouped by outcomes of ROC analyses as PET responders or non-responders. Optimal cutpoints were rounded to facilitate clinical use.

### Statistical analysis

Differences were tested using the non-parametric Mann–Whitney *U*-test. Correlations were tested using the Kendall rank correlation coefficient Tau B. Optimal thresholds were calculated by plotting receiver operator characteristics curves. Sensitivity, specificity, negative and positive predictive value for defined thresholds were estimated via Chi^2^ test. All computations relied on standard software (SPSS v24; IBM Corp, Armonk, NY, USA), setting significance at *p* < 0.05.

## Results

### Patient characteristics and treatment

The first 33 consecutive patients recruited at a single centre were included in this analysis. FDG-PET/CT was available pre- and post-induction therapy in 31 patients which were included in this analysis. Most tumors were located in the oropharynx (*N* = 15) of which ten were HPV positive. A total of 18 patients had a Tumor Proportion Score (TPS) per tumor cell (TC) percentage or a PD-L1-expression in the surrounding immune cells (IC Area) of at least 25% prior to initiation of ICIT. Patient characteristics are given in Table 1. Safety and efficacy of this ICIT treatment scheme have been previously reported [19].

**Table 1:** Characteristics of patient population (*n* = 31)

Variable	No of patients
Sex	
Male	27
Female	4
Tumor status	
cT1	2
cT2	4
cT3	6
cT4	19
Location	
Oropharynx, p16 positive	10
Oropharynx, p16 negative	5
Hypopharynx	10
Larynx	6
Grading	
G2	5
G3	16
Nodal status	
cN0	6
cN1	5
cN2	15
cN3	5
PD-L1-expression	
TPS/TC % < 25% + IC area < 25%	13
TPS/TC % < 25% + IC area ≥ 25%	11
TPS/TC % ≥ 25% + IC area < 25%	3
TPS/TC % ≥ 25% + IC area ≥ 25%	4
Therapeutic response	
pCR	17
ReTu	14

*HPV* human papilloma virus, *PD-L1* programmed death receptor-1 ligand, *TPS* tumor proportion score, *TC* tumor cell, *IC area* immune cell in tumor area, *pCR* Pathologic complete remission, *ReTu* residual tumor

### Re-biopsy results

Re-biopsy was performed in mean 26.2 ± 3.6 days after the initiation of ICIT. Re-biopsy revealed a pCR in 17 patients (55%) after ICIT, 14 patients showed ReTu (45%). There was no clear correlation between the timing of the re-biopsy and the occurrence of pCR (Ʈ_b_ = 0.17; *p* = 0.288). CD8 + cell density pre-ICIT was 1023.9 ± 821.8 cells per mm^2^, whereas CD8 + cell density post ICIT in patients with ReTu was 1294.2 ± 731.6 cells per mm^2^. There was a mean increase in CD8 + cell density post-ICIT of 29.5% ± 49.0%. In patients with pCR CD8 + cell density post ICIT was not estimated. We found no correlation between the measured metabolic parameters and the pre-ICIT and post-ICIT CD8 + cell density, e.g. correlation between pre-ICIT SUVmax PT and pre-ICIT CD8 + cell density was Ʈ_b_ = − 0.21 (*p* = 0.103). Further details can be found already published by our workgroup [19, 20].

### Pre-ICIT glucose metabolism

In all patients, PTs were identifiable by F18-FDG PET/CT imaging, with markedly elevated metabolic levels on average (SUVmax PT, 12.9 ± 5.1; TLG PT, 157.7 ± 151.7 cm^3^). Glucose metabolism was of similar extent at baseline in patients who failed to achieve pCR after ICIT (SUVmax PT: pCR, 11.8 ± 5.1 [95% CI 9.3–14.5]; ReTu, 14.1 ± 5.1 [95% CI 11.2–17.1]; *p* = 0.28). Baseline metabolic levels in patients with HNSCC of oropharynx (*n* = 15) did not differ significantly, regardless of human papilloma virus (HPV) status. Suspicious nodal metabolic activity at baseline was evident in 25 of 31 patients (80.7%), with an average SUVmax LN + of 9.7 ± 5.1 [95% CI 7.6–11.8]. The WB TLG was 229.3 ± 177.9 cm^3^ [95% CI 164.1–294.5]. An overview of quantitative measures is provided in Table 2.

**Table 2:** Overview of quantifiable FDG-PET/CT parameters, 95% Confidence Intervals in brackets

	Pre-ICIT			Post-ICIT		
	pCR	ReTu	*P*	pCR	ReTu	*P*
SUV_max_ PT	11.8 ± 5.1 [9.3–14.5]	14.1 ± 5.1 [11.2–17.1]	0.28	4.0 ± 2.3 [2.8–5.2]	9.8 ± 4.7 [7.1–12.5]	** < 0.01**
SUV_peak_ PT	9.8 ± 4.7 [7.4–12.2]	11.9 ± 4.6 [9.3–14.6]	0.20	3.6 ± 1.7 [2.6–4.5]	8.1 ± 4.4 [5.5–10.6]	** < 0.01**
MTV PT, cm^3^	17.2 ± 15.5 [9.3–25.2]	23.5 ± 17.7 [13.3–33.7]	0.26	5.2 ± 10.6 [– 0.2 to 10.7]	18.1 ± 26.1 [3.0–33.1]	** < 0.01**
TLG PT, cm^3^	118.2 ± 108.4 [62.4–173.9]	205.6 ± 184.8 [98.9–313.3]	0.12	21.8 ± 49.8 [– 3.8 to 47.4]	160.9 ± 347.4 [− 40.0 to 361.5]	** < 0.01**
SUV_max_ LN +	11.4 ± 5.6 [8.0–14.8]	7.9 ± 3.8 [5.4–10.3]	0.10	7.9 ± 4.5 [5.2–10.6]	6.6 ± 4.2 [4.0–9.3]	0.30
SUV_peak_ LN +	9.6 ± 4.8 [6.6–12.5]	6.1 ± 3.4 [3.9–8.3]	0.09	6.4 ± 3.3 [4.4–8.4]	5.2 ± 3.6 [3.0–7.6]	0.30
MTV WB	31.5 ± 21.0 [20.7–42.3]	30.0 ± 22.9 [16.8–43.2]	0.74	16.8 ± 17.8 [7.6–26.0]	23.8 ± 31.2 [5.7–41.8]	0.60
TLG WB	217.2 ± 143.0 [143.6–290.6]	244.1 ± 217.8 [118.3–369.8]	0.89	80.3 ± 100.4 [28.7–131.9]	198.2 ± 377.4 [− 19.7 to 416.1]	0.52
SUV_mean_ liver	2.41 ± 0.25 [2.27–2.54]	2.13 ± 0.36 [1.92–2.33]	**0.01**	2.35 ± 0.27 [2.19–2.50]	2.13 ± 0.44 [1.88–2.38]	0.06
SUV_mean_ ratio spleen/liver	0.82 ± 0.09 [0.77–0.87]	0.90 ± 0.08 [0.85–0.94]	**0.02**	0.84 ± 0.09 [0.79–0.90]	0.91 ± 0.10 [0.86–0.97]	**0.01**
SUV_mean_ ratio bone/liver	0.83 ± 0.13 [0.76–0.90]	0.93 ± 0.25 [0.79–1.08]	0.24	0.84 ± 0.16 [0.75–0.93]	0.91 ± 0.24 [0.77–1.04]	0.48
SUV_mean_ ratio lymph node/liver	0.59 ± 0.19 [0.49–0.69]	0.79 ± 0.29 [0.63–0.96]	**0.02**	0.64 ± 0.17 [0.54–0.74]	0.76 ± 0.21 [0.64–0.88]	0.08

Bold values are correct selected and indicate significant differences

*FDG-PET/CT* fluorodeoxyglucose—positron emission tomography/computed tomography, *ICIT* induction chemoimmunotherapy, *pCR* pathologic complete remission, *ReTu* residual tumor, *PT* primary tumor, *LNM* lymph node metastasis, *WB* whole body, *SUV* standardized uptake value, *MTV* metabolic tumor volume, *TLG* total lesion glycolysis

### Post-ICIT glucose metabolism

After ICIT, the average metabolic activity of PTs declined significantly (SUVmax PT, 6.6 ± 4.5 [95% CI 4.9–8.3]; TLG, 84.6 ± 242.2 cm^3^ [95% CI − 4.1 to 173.4]); and patients who achieved pCR showed significantly less metabolic activity than those who did not (SUVmax PT: pCR, 4.0 ± 2.3 [95% CI 2.8–5.2]; ReTu, 9.8 ± 4.7 [95% CI 7.1–12.5]; *p* < 0.001). The average SUVmax LN + after ICIT was 7.3 ± 4.3 [95% CI 5.5–9.1], with a TLG LN + of 60.6 ± 93.8 cm^3^ [95% CI 21.9–99.4]. The WB TLG after ICIT was 132.9 ± 265.9 cm^3^ [95% CI 35.4–230.5]. See Table 2 for other quantitative measures.

### Metabolic changes after ICIT

**Fig. 2:**
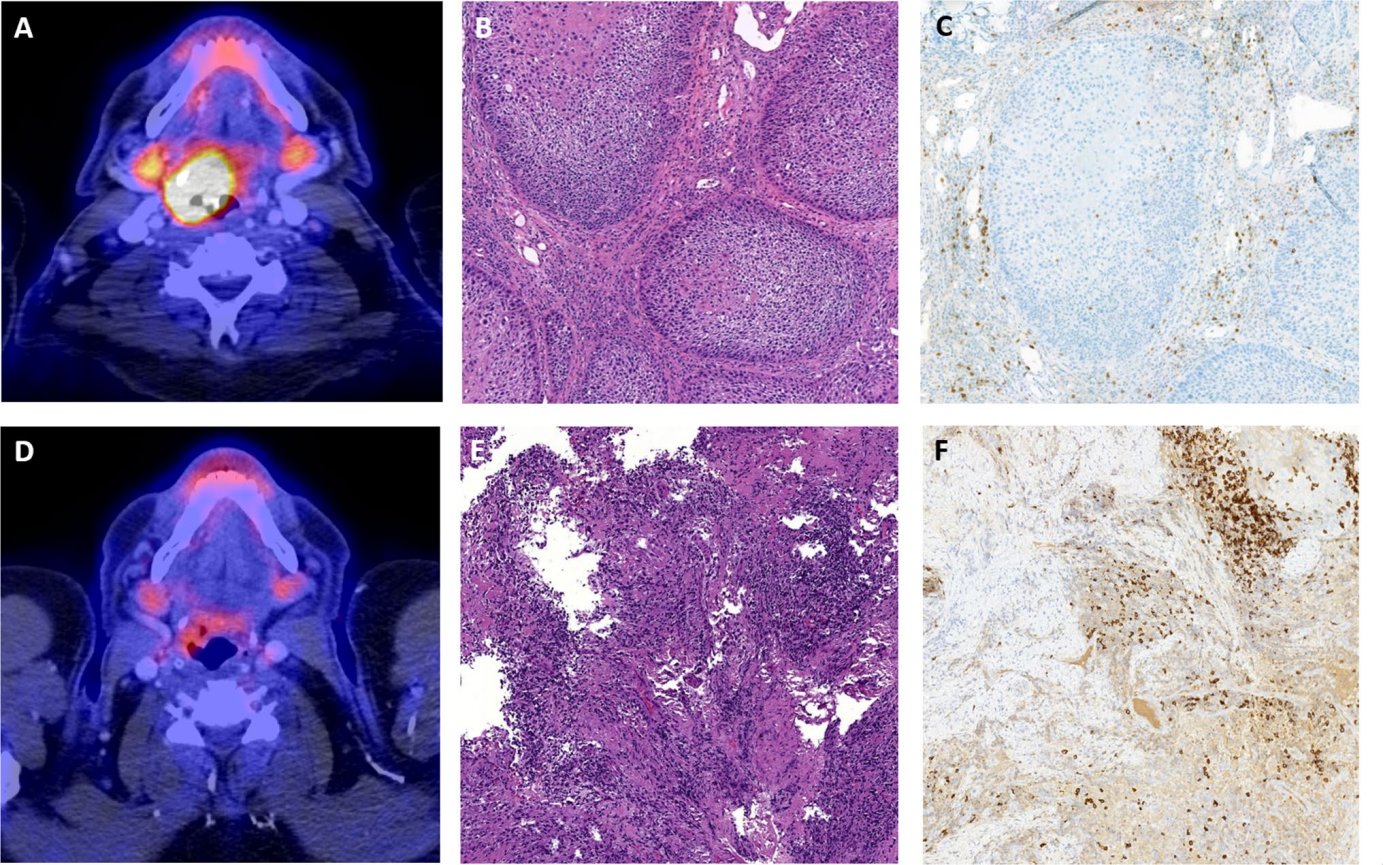
Upper row: Representative patient with (**A**) cT2 cN2c HNSCC of hypopharynx prior to induction chemoimmunotherapy. FDG-PET/CT records a high baseline metabolic activity high (SUVmax, 19.8). Histology shows a moderately to poorly differentiated non-keratinizing squamous cell carcinoma (**B**) HE staining with a low levels of stromal and intratumoral infiltration with cytotoxic T-Cells (CD8 immunohistochemistry) (**C**). Lower row: Same patient after ICIT: (**D**) marked decline in uptake by primary tumor (SUVmax, 2.8; ΔSUVpeak, 85.9%). Histology shows a densely packed lymphoid stroma in the HE stain (**E**) with CD8 positive lymphocytes after immunolabeling (**D**) but without evidence of viable tumor cells (**F**). *PT* primary tumor, *SUV* standardized uptake value, *HE* stain hematoxylin and eosin stain

In average, ΔSUVmax PT was 47.3 ± 31.8% [95% CI 35.7–59.0] and ΔTLG PT was 67.4 ± 46.4% [95% CI 50.4–84.4] following ICIT; and patients who achieved pCR showed a significantly greater decline in metabolic activity than those who did not (ΔSUVmax PT: pCR, 64.9 ± 15.8% [95% CI 56.8–73.1]; ReTu, 26.1 ± 33.7% [95% CI 6.6–45.5]; *p* < 0.001). Results for ΔSUVpeak PT and ΔTLG PT were similar. The ΔSUVmax LN + was 18.9 ± 33.5% [95% CI 5.1–32.8]. There was no significant difference in ΔSUVmax LN + whether or not pCR was achieved (pCR, 25.8 ± 29.4% [95% CI 7.7–42.9]; ReTu 12.2 ± 37.6% [95% CI − 11.7 to 36.1]; *p* = 0.44). The average ΔTLG WB was 56.5 ± 48.1%, patients achieving pCR showing significant greater change than those who did not (ΔTLG WB: pCR 68.7 ± 33.1% [95% CI 51.6–85.7%]; ReTu 41.7 ± 59.7% [95% CI 7.2–76.1%]; *p* = 0.044). Data on metabolic changes are given in Table 3. A representative patient example can be found in Fig. 2.

**Table 3:** Overview of percent change (Δ) in FDG uptake after induction chemoimmunotherapy (post-ICIT), shown by patient response, 95% Confidence Intervals in brackets

	pCR	ReTu	*P*
ΔSUV_max_ PT	64.9 ± 15.8 [56.8–73.0]	26.1 ± 33.7 [6.6–45.5]	** < 0.01**
ΔSUV_peak_ PT	60.4 ± 15.8 [52.3–68.6]	26.8 ± 35.8 [6.1–47.5]	**0.01**
ΔMTV PT, cm^3^	74.2 ± 21.8 [63.0–85.5]	38.0 ± 33.3 [18.8–57.2]	** < 0.01**
ΔTLG PT cm^3^	86.7 ± 15.1 [78.9–94.5]	44.0 ± 60.0 [9.3–78.6]	** < 0.01**
ΔSUV_max_ LN +	25.2 ± 29.4 [7.4–42.9]	12.2 ± 37.6 [− 11.7 to 36.1]	0.44
ΔSUV_peak_ LN +	25.2 ± 28.9 [7.7–42.6]	9.6 ± 46.3 [− 19.9 to 39.0]	0.38
ΔMTV WB	53.8 ± 35.0 [35.8–71.8]	36.8 ± 33.1 [17.7–55.9]	0.10
ΔTLG WB	68.7 ± 33.1 [51.6–85.7]	41.7 ± 59.7 [7.2–76.1]	**0.04**
ΔSUV_mean_ liver	0.5 ± 15.7 [− 8.6 to 9.5]	0.03 ± 14.2 [− 8.2 to − 8.2]	1.00
ΔSUV_mean_ ratio spleen/liver	− 3.6 ± 10.3 [− 9.5 to − 2.4]	− 2.2 ± 9.6 [− 7.8 to 3.3]	0.74
ΔSUV_mean_ ratio bone/liver	− 0.5 ± 19.4 [− 11.7 to − 10.7]	− 0.04 ± 24.1 [− 13.9 to 13.8]	0.80
ΔSUV_mean_ ratio lymph node/liver	− 9.8 ± 20.7 [− 21.8 to 2.1]	− 0.02 ± 18.9 [− 11.0 to − 10.9]	0.22

Bold values are correct selected and indicate significant differences

*pCR* pathologic complete remission, *ReTu* residual tumor, *PT* primary tumor, *LN* +  lymph node metastasis, *WB* whole body, *SUV* standardized uptake value, *MTV* metabolic tumor volume, *TLG* total lesion glycolysis

### Metabolic activity of lymphatic and hematopoietic tissue

Mean FDG uptake values in spleen, bone, and a representative region 7 mediastinal node were fairly consistent in patients overall, with no apparent changes in metabolic activity levels before and after ICIT (see Table 2). Liver uptake was significantly greater in patients who achieved pCR than in those who did not (SUVmean: pCR, 2.4 ± 0.3 [95% CI 2.3–2.5]; ReTu, 2.1 ± 0.4 [95% CI 1.9–2.3]; *p* = 0.01), even before initiation of ICIT. However, similar results were noted after ICIT. There were no significant changes in background activity levels after ICIT, as shown in Table 3.

### Receiver operating characteristic (ROC) analyses

**Fig. 3:**
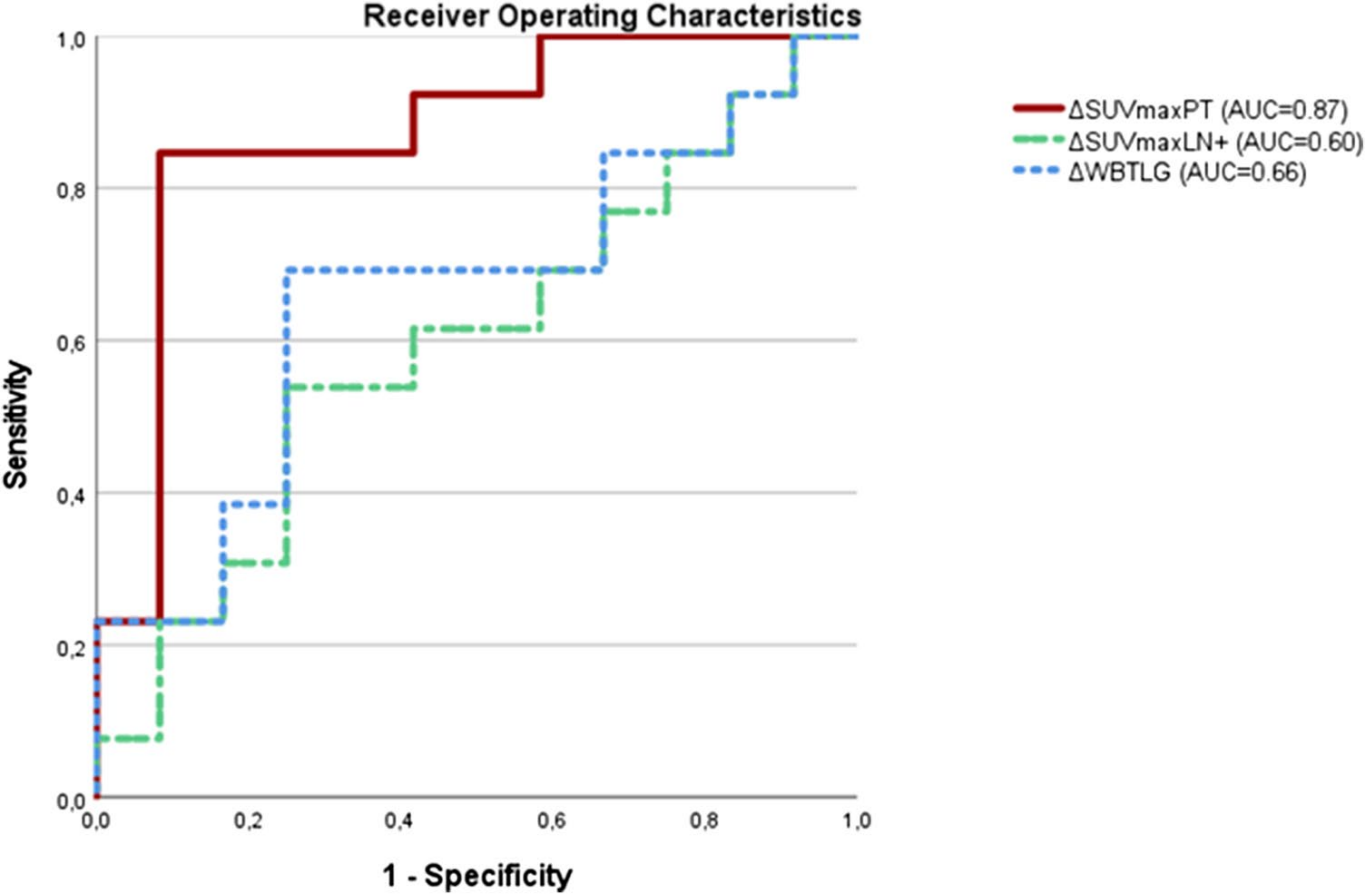
Receiver operating characteristic (ROC) curve plotted for complete remission in subset of patients with nodal metastasis (*n* = 25)

In ROC analysis of pCR, ΔSUVmax PT yielded the highest AUC (0.89), with an optimal cutpoint (OC) of 0.56 for decline in tumor metabolism (sensitivity, 0.88; specificity, 0.93). Results for ΔSUVpeak PT (AUC = 0.83; OC, 0.53; sensitivity, 0.82; specificity, 0.71) and ΔTLG PT (AUC = 0.85; OC, 0.90%; sensitivity, 0.77; specificity, 0.86) were similar. ΔTLG WB showed the lowest performance (AUC = 0.714; OC, 0.75; sensitivity, 0.71; specificity, 0.79) (Fig. 3).

In terms of ReTu, measuring residual PT activity yielded the best ROC outcome (SUVmax PT Post-ICIT: AUC = 0.908; OC, 6.0; sensitivity, 0.86; specificity, 0.88).

ROC analysis indicated no apparent correlation between histologic response and activity levels or changes thereof in LN + or other lymphatic and hematopoietic organs pre- or post-ICIT.

### Subgroup analysis

To investigate the clinical applicability of ROC analyses, we established the following cutpoints for declines in FDG uptake by PET responders, grouping patients according to various standards: (1) European Organization for Research and Treatment of Cancer (EORTC) PET criteria [21] → ΔSUVmax PT ≥ 0.25; (2) Positron Emission Tomography Response Criteria in Solid Tumors (PERCIST) defined by Wahl et al. [22] → ΔSUVpeak PT ≥ 0.30; and (3) ROC analysis → ΔSUVmax PT ≥ 0.50, ΔSUVmax PT ≥ 0.60, and ΔTLG WB ≥ 0.75. We also combined the two best quantitative parameters at set thresholds, namely ΔSUVmax PT ≥ 0.50 and SUVmax PT Post-ICIT ≤ 6, obtaining high-level specificity and NPV (both, 0.93) in predicting pCR. All sensitivity, specificity, NPV, and PPV data are found in Table 4.

**Table 4:** PET responses at various thresholds in testing for pCR (17 of 31 patients)

	PET responders (*n*)	Sensitivity	Specificity	NPV	PPV
ΔSUVmax PT ≥ 25.0% (EORTC)	23	0.94	0.50	0.70	0.87
ΔSUVpeak PT ≥ 30.0% (PERCIST)	24	0.94	0.57	0.73	0.88
ΔSUVmax PT ≥ 50.0%	20	0.88	0.64	0.75	0.81
ΔSUVmax PT ≥ 60.0%	14	0.76	0.93	0.93	0.76
ΔTLG WB ≥ 75.0%	15	0.80	0.79	0.82	0.76
ΔSUVmax PT ≥ 50.0% + SUVmax PT post-ICIT ≤ 6	15	0.82	0.93	0.93	0.81

*PET* positron emission tomography, *EORTC* European organization for research and treatment of cancer, *PERCIST* positron emission tomography response criteria in solid tumors, *NPV* negative predictive value, *PPV* positive predictive value, percentage decrease (Δ), *PT* primary tumor, *LNM* lymph node metastasis, *WB* whole body, *SUV* standardized uptake value, *TLG* total lesion glycolysis

## Discussion

The current single-center analysis of the prospective phase II CheckRad-CD8 trial found a pCR rate of 55%. This is comparable to the interim analysis of this endpoint of the whole multi-center study [19]. The current single-center analysis focuses on the question if the pCR can be identified by FDG-PET/CT.

To our knowledge, this the first study reporting on the use of FDG-PET/CT for monitoring early responses to combined ICIT in patients with locally advanced HNSCC. However, other researchers have similarly evaluated responses to IC in this setting. In 2013, Kikuchi et al. published a study on the predictive value of early metabolic response after neoadjuvant chemotherapy. They defined PET response as a decline in SUVmax ≥ 55% or a residual SUVmax ≤ 3.5 at PT sites, identifying early PET response as an independent prognostic factor for local tumor control and disease-specific survival. Our own study group has recently published a paper on metabolic responses after one cycle of IC, confirming the results of Kikuchi et al. [10, 11]. In this work, a decline in SUVmax of at least 60% corresponded with better local tumor control (LC) and OS, whereas insufficient FDG-PET responses were linked to high local recurrence rates after definitive CRT or surgery due to initial IC failure.

On the other hand, it is not entirely clear whether if FDG-PET/CT is suitable for monitoring ICIT responses. Past studies aimed at other malignancies have shown its erratic diagnostic accuracy when used for this purpose, one being a report by Gilles et al in which a small patient population received interferon-alpha for renal cell carcinoma [23]. They found no correlation between post-therapeutic changes in FDG uptake and progression-free survival (PFS) or OS. Furthermore, because immunotherapy triggers local inflammation within tumorous areas, there are several accounts of metabolic pseudoprogression on PET [24–26]. Inhibitors of the PD-1/PD-L1 pathway are known to induce a systemic immune response leading to extensive immunologic changes measurable in the peripheral blood [27]. As recently published, such systemic immunological changes can lead to misclassification of metabolic responses in up to one-third of patients monitored by FDG-PET/CT after PD-L1-blockade for non-small cell lung cancer [28].

In our patient group, there was a high concordance between metabolic activity and clinical remission. In the future this might hold the possibility to replace invasive biopsy to evaluate treatment response and allow an appropriate patient selection for radio-immunotherapy. The value of patient selection for definitive radio-chemotherapy based on metabolic response has already been proven in the concept of conventional induction chemotherapy [6]. Probably due to the limited number of patients studied, we found no obvious cases of metabolic pseudoprogression. We identified one patient with a significant increase in metabolic activity on post-ICIT scan (ΔSUVmax PT = − 49.6%), fulfilling the conditions of an unconfirmed progressive metabolic disease (UPMD) as defined by the recently published iPERCIST-criteria [29]. Histologic assessment confirmed an insufficient response to ICIT. The prospect of metabolic pseudoprogression in our study setting remains uncertain, as only the basline and one subsequent PET was performed. One could speculate that when induction chemotherapy and immunotherapy are combined, the decrease in FDG uptake due to the cytotoxic effects of IC may be overridden by a potential inflammatory increase in FDG uptake. This is supported by our post-ICIT findings, namely median residual SUVmax PT post-ICIT of 5.3 and median decline of 56.7%, and the analogous reporting of residual FDG uptake (SUVmax, 5.8) in our own group of patients with HNSCC after IC alone [6]. Additional studies are still needed to clarify this point. Histologic pCR (vs ReTu) was associated with significantly greater decline in PT metabolic activity measured by FDG-PET/CT (*p* < 0.01).

In the ROC analyses, changes in FDG uptake yielded high AUCs (up to 0.89) in predicting pCR, whereas residual activity (SUVmax PT post-ICIT ≥ 6) correctly predicted ReTu (AUC = 0.91) sensitivity = 0.86; specificity = 0.88). The combination of both ΔSUVmax PT ≥ 0.5 and SUVmax PT post-ICIT ≤ 6 enabled prediction of pCR after ICIT with a high level of diagnostic confidence (sensitivity, 0.82; specificity, 0.93; NPV, 0.93; PPV, 0.81).

All quantitative parameters investigated, including SUVmax, SUVpeak, MTV, and TLG, performed comparably in our study and are viable indices for therapeutic monitoring. Given that EARL criteria v1.0 (accredited for PET reconstruction) were applied to our analysis, allowing comparisons of SUVmax determinations across various institutions and PET systems, we concentrated on reporting of SUVmax. However, SUVpeak and TLG may further improve such comparability. Unfortunately, FDG uptake in LN + or in healthy lymphatic and hematopoietic tissue as a surrogate measure of immunotherapy response is not a valid approach. Our group has already published similar results for patients after IC, and there was simply no correlation between therapeutic response and FDG uptake in nodal metastases [7].

At this juncture, we have reported early pathologic responses after ICIT in a limited number of patients with HNSCC. No data on long-term follow-up or OS is available so far to assess the prognostic utility of FDG-PET/CT in these patients. Additional studies focusing on clinical outcomes are essential in addressing this issue.

## Conclusion

In patients with locally advanced HNSCC, quantifying residual FDG uptake levels of PTs and corresponding percent changes allows the identification of histological complete response after a single cycle of ICIT. However, background activity within lymphatic and hematopoietic organs and quantifiable FDG uptake in nodal metastases are no valid measures.
